# Critical review on quality of methodology and recommendations of clinical practice guidelines for peri-implantitis

**DOI:** 10.1186/s12903-023-02904-4

**Published:** 2023-03-31

**Authors:** Qiaofeng Yin, Jingyi Liang, Yuhang Zhang, Canxiong Chen, Weiming Yu, Xiaoyi Wang, Jianxin Ji

**Affiliations:** 1 Department of Stomatology, The Seventh Affiliated Hospital, Southern Medical University, 28 Desheng Liguan Road, Foshan, 528244 Guangdong P.R. China; 2 Department of Stomatology, The First Affiliated Hospital of Guangzhou Medical University, 151 Yanjiang Road, Guangzhou, 510120 Guangdong P.R. China; 3 National Center for Respiratory Medicine, State Key Laboratory of Respiratory Disease & National Clinical Research Center for Respiratory Disease, Guangzhou Institute of Respiratory Health, The First Affiliated Hospital of Guangzhou Medical University, Guangzhou, 510120 Guangdong P.R. China

**Keywords:** Peri-implantitis, Peri-implant disease, Guideline, AGREE II

## Abstract

**Background:**

Peri-implantitis is of high prevalence with the popularity of dental implants nowadays. Guidelines or consensus have been developed in succession, and we are little-known about their quality. The objective of this study is to evaluate the methodological quality of these guidelines and analyze the consistency of the clinical recommendations.

**Methods:**

We searched for guidelines or consensus on prevention, diagnosis, and/or treatment of peri-implantitis through PubMed, Web of Science, Cochrane Library until January 15th, 2022. In addition, we also searched the websites of the American Dental Association, International Team for Implantology, FDI World Dental Federation, and some guideline collection databases. Appraisal of Guidelines for Research & Evaluation II methodological quality instrument was used to assess the selected guidelines. Furthermore, we described the consistency of recommendations across the included guidelines.

**Results:**

In total, 15 guidelines were included. The mean values of the six domains score all below 50%. The mean scores of Applicability were lowest (mean:15%, range:4–29%). As to the overall quality, eleven (73%) were recommended after being modified, and four (27%) were not recommended. Among the clinical recommendations, 53 (67.09%) are for treatment of peri-implantitis, 13 (16.46%) for monitoring issue, 7 (8.86%) for diagnosis, 3 (3.80%) for the disease prevention.

**Conclusions:**

Improving methodology quality and strengthening clinical evidence is essential in the future guideline development in a range of disciplines for improving the treatment effectiveness of people with peri-implantitis. And there is a lack of integrated guidelines in the case of the COVID-19 pandemic.

**Supplementary Information:**

The online version contains supplementary material available at 10.1186/s12903-023-02904-4.

## Introduction

Peri-implantitis is a pathological condition occurring in tissues around dental implants, characterized by inflammation in the peri-implant connective tissue and progressive loss of supporting bone [1, 2]. As we know, a history of periodontitis, dental plaque, poor oral hygiene, smoking, alcohol consumption, and diabetes are the risk factors for peri-implantitis [3]. With the popularity of dental implants, a high prevalence has been shown in serials of epidemiology survey of peri-implantitis, ranging from 8 to 60% [4–8]. If peri-implantitis is not treated timely, its progression normally follows a non-linear and accelerating pattern [9]. Hence, the importance of prevention and treatment of peri-implantitis should be attached. Clinical practice guidelines(CPGs) are the compass for medical behavior [10], of which quality affects the treatment effectiveness. Appraisal of Guidelines for Research and Evaluation (AGREE) II tool is a widely accepted instrument for guideline development appraisal [11], evaluating the quality and reporting of practice guidelines using 23 items across six domains. In recent years, clinical evidence is continually emerging on prevention, diagnosis, treatment along with the development of dental implantology, and clinical guidelines or consensus on peri-implantitis are published frequently. To date, their quality remains unknown. In this study, we conduct an appraisal on the methodological quality of published guidelines, and furtherly extract the clinical recommendations on several topics (i.e., prevention, diagnosis, and treatment), providing an evidence-based reference to clinicians to reasonably follow CPGs and to the medical community to optimize the development in the future.

## Methods

### Study design

We conducted an assessment of the quality of CPGs on peri-implantitis with the AGREE II instrument. The study protocol was registered in PROSPERO (CRD42021285546) and the results are reported in line with the Preferred Reporting Items for Systematic Reviews and Meta-Analyses (PRISMA) statements (Supplementary material part 3).

### Searches strategy

We searched the public databases including PubMed, Web of Science, Cochrane Library, and websites of relevant organizations (e.g., American Dental Association, International Team for Implantology, FDI World Dental Federation etc.) and grey databases (e.g. WHO guideline, Guidelines International Network, Scottish Intercollegiate Guidelines Network etc.) up to January 15th 2022. Reference lists searching and experts consulting were conducted to ensure a comprehensive review. The search words included “peri-implantitis”, “peri-implant disease”, “guideline”, “recommendation” and “consensus”, and more details of search strategy could be seen in Supplementary material part 1.

### Guideline selection

We included documents that focused on the diagnosis or management of peri-implantitis. The documents should be developed by international, national health organizations or stomatology societies. Only documents in English would be eligible. Keeping up with these criteria, two researchers screened the documents respectively and disagreements were resolved by discussion and consensus.

### Data extraction

Where available, the following information from each article was extracted using a pre-set data extracted form, including title, acronym of the guideline, publication date, country applied, region, type of guidelines, issuing society full name, type of publication, development method, evidence-grading system, strength of recommendations, quality of evidence, developers and number of developed organizations and so on.

### Guideline Quality Assessment

Four experienced researchers independently appraised each eligible guideline by using the AGREE II instrument, which provides an objective evaluation to assess the quality of guidelines. This tool consists of six domains (‘Scope and purpose’, ‘Stakeholder involvement’, ‘Rigorous of development’, ‘Clarity of presentation’, ‘Applicability’, and ‘Editorial independence’) and two overall guidelines assessment. According to the instruction, a total score more than 60% will be determined as “recommended”, a score range 30% from 60% as “recommended with modification” and below 30% as “not recommended”.

### Clinical recommendations classification

We carefully read each document and extract clinical recommendations related to peri-implantitis, and the strength of recommendation and quality of evidence subsequently. The extracted content mainly includes the recommendations on the prevention, diagnosis, and treatment of peri-implantitis. In terms of The criteria for classifying the strength of recommendation and quality of evidence is generally different for most the guidelines. To solve this problem, we use a new comprehensive classification criterion and an additional word file shows this in more detail(Supplementary material part 2), which we can redefine and compare the recommendations.

### Data analysis

We conducted a descriptive analysis on the general characteristics of the included guidelines. For each item, the minimum score was 1 (the lowest score) multiplied by the number of reviewers, and the maximum score was 7 (the highest score) multiplied by the number of reviewers. Domain scores were calculated by adding the scores of individual domain items and scaling the total as a percentage of the maximum possible score for that domain: [(obtained score) – (minimum score)] / [(maximum score) – (minimum score)]. The domain scores are presented per domain per CPG as percentages with the mean score for all CPGs.

## Results

### Characteristics of eligible guidelines

**Fig. 1:**
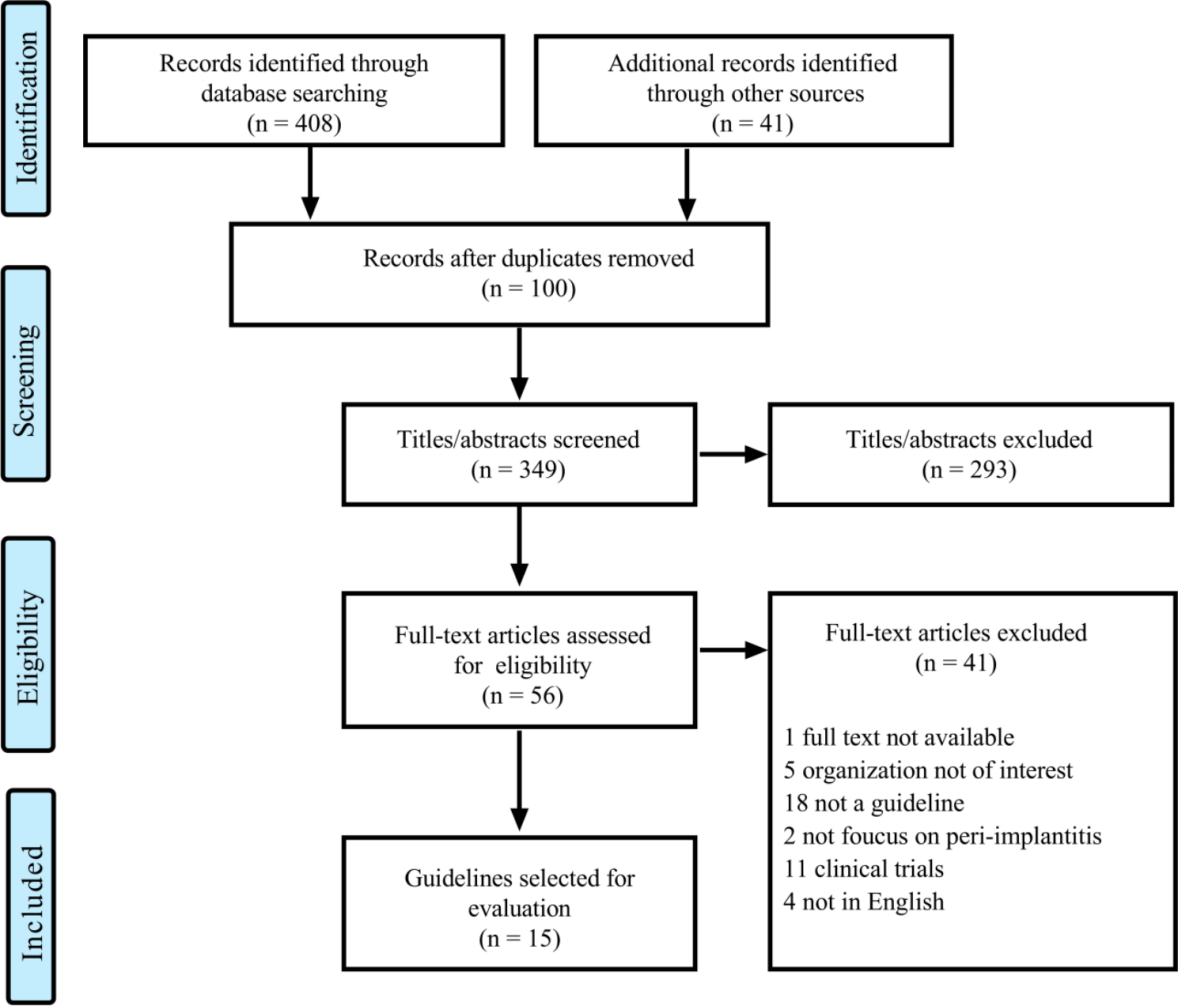
**Flow diagram for the selection of guidelines.** We divided the the selection of eligible guidelines into four parts, and the included and excluded details are listed

**Fig. 2:**
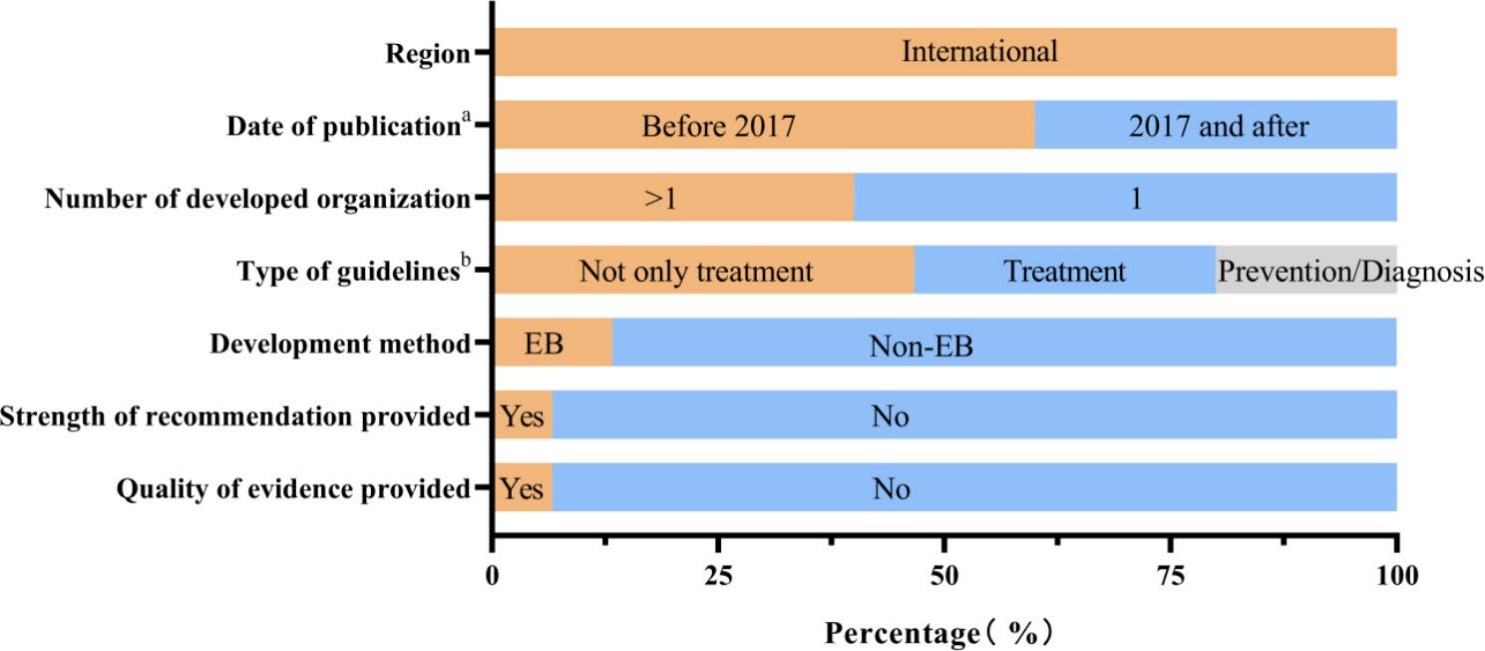
**General characteristics of eligible guidelines.** (a) Classification of Peri-implantitis Diseases and Condition Case are described in the 2017 World Workshop on the. (b) Type of guideline was divided into three parts according to the content

Fifteen guidelines met the inclusion criteria (Fig. 1). Six (40%)[12–17] were published after 2017. All were developed by international organizations and only one of them [18] was a self-proclaimed guideline. Thirteen (87%) were developed in an evidence-based approach. None of them has developed an updated version. All were developed by the medical society, and six [12, 13, 19–22] (40%) were developed by more than one organization. Nearly half of them involved topics not limited to management. Two [20, 23] were exclusively focused on prevention, one [12] on diagnosis and five [13, 14, 16, 19, 21] on management. (Fig. 2) Only one [12] (6.67%) provided the strength of recommendation or the quality of evidence and more details could be seen in Table. S1.

### Quality Assessment of Guidelines

#### Totality

**Fig. 3:**
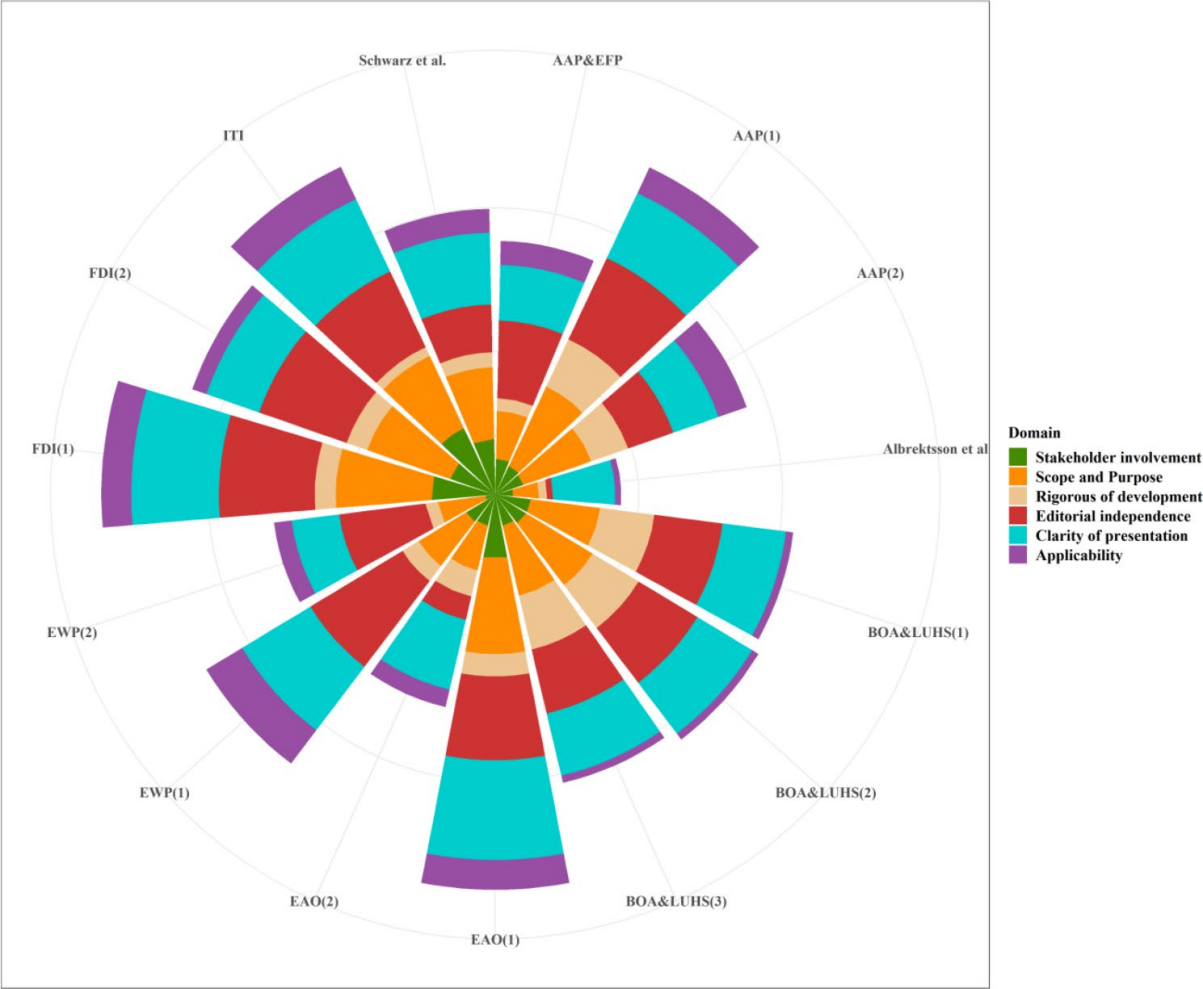
**Scores in six domains with AGREE II tool.** Detail score of guidelines were listed in Supplementary Table 2. Acronym of the guideline is listed in outside circle, and radius length of the circle represents the different domain scores

In AGREE II appraisal, *Editorial Independence* (mean: 49%, range: 4–75%), *Scope and Purpose* (mean: 48%, range: 18–67%) and *Clarity of Presentation* (mean: 48%, range: 33–69%) had higher average scores. *Stakeholder Involvement* (mean: 28%, range: 7–51%) had lower average scores. *Rigor of Development* (mean: 20%, range: 6–39%) *and Applicability* (mean: 15%, range: 4–29%) had the lowest average scores. As for the overall evaluation, eleven (73%) were recommended with modification, and four (27%) were not recommended. Overall quality as assessed by the AGREE II tool was dis-satisfactory (Fig. 3). Scores of four appraisers was considered as highly consistent (Intraclass correlation coefficient, ICC: 0.969, 95%CI: 0.964–0.974).

### Recommendations

A total of 79 recommendations related to peri-implantitis were extracted from 15 guidelines. Only 2 of them (2.53%) provided the level of evidence (low:2), and 1 (1.27%) provided with the strength of recommendation (weak:1) (Table S1). Among the clinical recommendations, 53 (67.09%) are for treatment of peri-implantitis, 7 (8.86%) for diagnosis of peri-implantitis, 3 (3.80%) for the disease prevention (Table 1).

**Table 1:** Recommendations for the management on peri-implantitis†

Topics	Recommendation	Supporting guidelines	Number of recommendations	Strength of recommendation#	Quality of evidence‡
Prevention	It is recognized that secondary prevention of peri-implantitis poses unique challenges that may only be partially addressed by routine supportive periodontal care programs.	EWP(2)	1	Ungraded	Ungraded
	Self-performed hygiene care or professional maintenance program (e.g. proper plaque control) have positive effect on preventing peri-implant mucositis proceeding into peri-implantitis	ITI, BOA&LUHS(2), BOA&LUHS(3)	3	Ungraded	Ungraded
	Caution should be used for implants in Sjögren’s Patients and diabetic patient which may have an increased risk for peri-implantitis.	EWP(1), BOA&LUHS(1)	2	Ungraded	Ungraded
Diagnosis	A diagnosis of peri-implantitis is given in the presence of mucositis in conjunction with progressive crestal bone loss	ITI	1	Ungraded	Ungraded
	When clinical signs suggest the presence of peri-implantitis, the clinician is advised to take a radiograph of the site to confirm the diagnosis.	EWP(1), BOA&LUHS(3)	2	Ungraded	Ungraded
	Diagnosis of peri-implantitis requires: • Presence of bleeding and/or suppuration on gentle probing. • Increased probing depth compared to previous examinations. • Presence of bone loss beyond crestal bone level changes resulting from initial bone remodeling.	AAP&EFP, FDI(2)	2	Ungraded	Ungraded
	In the absence of previous examination data, diagnosis of peri-implantitis can be based on the combination of: • Presence of bleeding and/or suppuration on gentle probing. • Probing depths of ≥ 6 mm(or ≥ 4 mm). • Bone levels ≥ 3 mm (or ≥ 5 mm) mapical of the most coronal portion of the intraosseous part of the implant	AAP&EFP, BOA&LUHS(3)	2	Ungraded	Ungraded
Treatment	A regular maintenance program may be needed for the long-term management of peri-implantitis lesions.	ITI	1	Ungraded	Ungraded
	Pretreatment phase including: i. Thorough assessment and diagnosis ii. Reduction of risk factors for peri-implantitis; in particular poor oral hygiene, prostheses that prevent adequate access for plaque control, to-bacco use, presence of periodontal diseases, and systemic diseases that may predispose to peri-implant disease ii. If required, prosthesis removal and adjustment/replacement	ITI	1	Ungraded	Ungraded
	Nonsurgical debridement focused on maximal removal of biofilm, with or without antimicrobials	ITI, EAO(1)	2	Ungraded	Ungraded
	The clinician should consider implant removal as a treatment option. Factors influencing this decision may include the severity of the peri-implantitis lesion, the position of the implant, the surrounding tissues, or when the treatment outcomes are likely to be unsatisfactory.	ITI, EAO(1), BOA&LUHS(3)	3	Ungraded	Ungraded
	If non-surgical treatment does not resolve the peri-implantitis lesion or arrest progressive bone loss, surgical therapy may be considered.	EAO(1), FDI(2)	2	Ungraded	Ungraded
	Proper pre- and postsurgical hygiene maintenance phases and successful implant surface decontamination are mandatory for successful surgical regenerative procedure.	Albrektsson et al., EWP(1), BOA&LUHS(2)	3	Ungraded	Ungraded
	Surgical augmentative peri-implantitis therapy results in improved clinical and radiographic treatment outcomes, which is considered to be superior to non-surgical therapy in resolving peri-implantitis.	FDI(1), EAO(1), AAP(2), FDI(2), BOA&LUHS(2)	5	Ungraded	Ungraded
	Surgical regenerative treatment might be chosen for intrabony defect reconstruction, whereas non-regenerative approach and implantoplasty of the supracrestal implant component is recommended.	FDI(1), BOA&LUHS(2)	2	Ungraded	Ungraded
	Implant surface scaling plus antimicrobial photodynamic therapy versus implant surface scaling for the treatment of peri-implantitis is recommended compared with scaling and root planing.	AAP(1)	1	Weak	Low
Others	**Monitoring**				
	Regular assessment of peri-implant health is recommended during support periodontal treatment (SPT) to identify disease at an early stage.	ITI, BOA&LUHS(3)	3	Ungraded	Ungraded
	The presence of purulent exudate in combination with clinically significant progressing Crestal bone loss (CBL) necessitates therapeutic intervention.	Albrektsson et al., EAO(1), EWP(1), EWP(2), AAP&EFP	5	Ungraded	Ungraded
	Evaluate iatrogenic factors that might have caused the disease, including cement remnants, malpositioning of the implant, inadequate restoration-abutment seating, and overcontouring of the reconstruction that disturbs proper plaque control should be evaluated.	BOA&LUHS(3)	1	Ungraded	Ungraded
	Clinical monitoring should be performed on a regular basis and supplemented by appropriate radiographic evaluation as required.	Albrektsson et al., ITI, AAP&EFP	3	Ungraded	Ungraded
	**Qualifications**				
	Training of dental team professionals should include diagnosis and management of peri-implant disease.	ITI	1	Ungraded	Ungraded
	**Biomarker**				
	Evidence regarding biomarkers and enzymes in peri-implant crevicular fluid (PICF) as possible predictors for peri-implantitis are very limited.	BOA&LUHS(3)	1	Ungraded	Ungraded

†: The full names of the abbreviation of guidelines are as same as those in Supplementary Table 1

‡: Strength of recommendation and quality of evidence were harmonized according the composite grading system shown in Supplementary material part 2

## Discussion

Nowadays, dental implantology has become more frequent to treat aesthetic and functional problems induced by natural tooth loss. and the incidence of peri-implant diseases also increased correspondingly. As an irreversible disease, peri-implantitis used to result in the implant loss. Prevention, diagnosis, and treatment are of equal importance in peri-implantitis management. To our knowledge, this is the first study that critically appraise the scientific evidence and recommendations of guidelines or consensus on peri-implantitis.

In terms of 15 consensus or guidelines in peri-implantitis, median scores for six AGREE II domains (*Scope and Purpose, Stakeholder involvement, Clarity of Presentation, Rigorous of development, Applicability, and Editorial Independence*) were less than 50%. Considerable discrepancies between documents were seen as reflected by the wide IQRs. As the result shows, the score was lowest in the *Applicability*, indicating there is still a distance to take the evidence provided into practice. Regarding the score of *Rigor of Development scores* and *Stakeholder Involvemen*t were second and third lowest respectively, there is a need to improve the methodological quality and include patient’s views in the further development. Besides, only one guideline was given the strength of the recommendations and quality of evidence. More detailed, 98.73% of guidelines did not given the recommendation strength, and 97.46% were ungraded quality in view of currently insufficient evidence. Even provided, the recommendation was based on the simple review of several observational studies, which may be of poor quality.

In the prevention section, the recommendations are relatively uninformed. It’s well recognized that self-performed hygiene care or professional maintenance program (e.g. proper plaque control) have a positive impact on preventing peri-implant mucositis from proceeding into peri-implantitis. Undoubtedly, patients play an important role in good oral hygiene carried out consistently and thoroughly. However, it’s vital that the regime demonstrated by hygienist and therapist or nurse oral health educator is easy, achievable and simple so that it becomes embedded in the patient’s daily routine [24]. In additional, EWP(1)[25] and BOA&LUHS(1)[20] call for attention should be paid to implants in Sjogren’s patients and diabetic patient respectively. While Daniel Almeida. et al [26] revealed the contrary conclusion that dental implant therapy in Sjogren’s patients seems to present high implant survival rate, so a greater number of prospective studies in the future is essential to support more robust conclusions.

The existing guidelines consistently suggest monitoring necessitates along the presence of dental implant. During support periodontal treatment (SPT), a regular assessment of peri-implant health is recommended to identify disease at an early stage [18, 22], and radiographic evaluation is an appropriate supplement as required [12, 18, 27]. Iatrogenic factors that might have caused the disease, including cement remnants, mispositioning of the implant, inadequate restoration-abutment seating, and over contouring of the reconstruction should be evaluated. Notably, once the presence of purulent exudate in combination with clinically significant progressing Crestal bone loss (CBL), several studies [12, 23, 25, 27, 28] jointly agreed that it’s time for a therapeutic intervention. However, in above guidelines, there is no clear guidance on how to conduct monitoring. It is crucial to be thorough and methodical when monitoring peri-implant tissues. First and immortally, a recording of an initial baseline assessment and taking of radiographs is suggested. After methodical clinical assessment that early careful diagnosis and spotting the clinical markers to assess the presence and severity of inflammation around the implant at regular review appointments and further radiographs should be made following if there is a indication. The presence of biofilm, inflammation of the peri-implant tissues, increase in peri-implant probing depth, bleeding on probing, suppuration from the peri-implant pocket, mobility and resulting radiographic changes are important to be noted.

Diagnosis of peri-implantitis depend heavily on radiological examination. The diagnosis basically divided into two conditions based on whether there is previous examination data.

It’s highly agreed by AAP&EFP [12] and BOA&LUHS(3)[22] that if there is a previous examination, bleeding and/or suppuration on gentle probing, and increased probing depth and bone loss indicated the occurrence of peri-implantitis. In contrast, their opinion differed in the diagnostic value of probing depth and bone loss without an initial value recorded. There is no consensus on the absolute value on probing depth represents the diagnosis of peri-implantitis, while the diagnostic value (probing depth > 5 mm) is an indicator the do radiology examination to evaluate possible bone loss. As to bone loss, the thresholds to diagnose peri-implantitis vary from study to study: Padial-Molina et al.[29]suggested that bone loss > 2 mm indicated peri-implantitis, while Misch et al.[30] suggested the threshold of > 4 mm. Furtherly, Ata-Ali et al.[31]suggested the classification of peri-implantitis should be based on the amount of marginal bone loss beyond biological bone remodeling(Stage I: ≤ 3 mm; Stage II: > 3 mm but < 5 mm; Stage III: ≥ 5 mm; Stage IV: ≥ 50% of the implant length). In terms of the uniform cutoff value, meta-analysis is called for to a scientific standard for further guideline development.

It’s well-recognized among current guidelines that as to treatment of peri-implantitis, surgical augmentative peri-implantitis therapy leads to improved clinical and radiographic treatment outcomes, which is considered to be superior to non-surgical therapy in resolving peri-implantitis [14, 16, 17, 21, 28]. Mario Roccuzzo et al. have conducted serval long period cohort studies revealing the long-term efficacy of reconstructive treatment followed by SPT on peri-implantitis [32–34]. Meanwhile, the invasiveness of the reconstructive procedures for peri-implantitis lesions is worthy of attention and should be properly evaluated for further guidance development [35]. Therefore, clinicians should carefully evaluate the severity of peri-implantitis, and facilitate therapy step by step. Bacterial induced inflammation is initially treated non-surgically with the use of locally administered treatments and adjuncts [18, 28]. If the peri-implantitis lesion or arrest progressive bone loss didn’t resolve by non-surgical treatment, surgical therapy may be considered. Whatever, oral hygiene is of great importance. Proper pre- and postsurgical hygiene maintenance phases and successful implant surface decontamination are mandatory for successful surgical regenerative procedure [25, 27, 28]. Besides oral hygiene, all underlying dental disease should be treated or stabilized before implant placement. And there are some localized predisposing factors, including the presence of plaque pathogenic biofilm and its endotoxins, prosthetic design and occlusal overload, retained cement, soft tissue quality and quantity and salivary reduction in patients with xerostomia.

Our study systematically searched and summarized the available guidelines and consensus on peri-implantitis, and furtherly appraise the methodology quality with AGREE II. As our result shown, the overall quality was unsatisfactory, and especially in Applicability. As to the recommendations on peri-implantitis management, it covers prevention, diagnosis and treatment, which serves as a clear guidance for clinician to daily practice, while the recommendation strength and quality needs to improve. However, there are inevitable downsides to this study. Firstly, some guidelines published not by medical organization or in English were excluded, which may result in a bias towards total situation. Secondly, although we have conducted a comprehensive research, some documents may be updated during the process of appraisal. Since the COVID-19 epidemic is still ongoing, COVID-19 infection may exacerbate peri-implantitis or affect patients’ follow-up [36]. The protocol of follow-up and continuous monitoring of patients with peri-implantitis during the COVID-19 period should be added to the guidelines, aiming to avoid exacerbation of the disease or implant failure.

Although Osseointegration and implant therapy, peri-implant inflammation have been put up respectively in the 60 and 90 s in the last century, peri-implantitis seems to be a “new disease” based on the quality of current guidelines. To further improve the quality of peri-implantitis guidelines, it is necessary to do a good job of investigating real clinical needs. Clinical questions arise mainly from surveys of guideline users, especially front-line clinicians, or current literature (related guidelines, systematic reviews, or clinical studies). The source, quantity, and composition of clinical problems not only determine the length of the guidelines and the content of recommended opinions but also influence the dissemination and application of the guidelines. When the clinical problems are highly relevant to first-line clinicians and the problems are clearly expressed, the implementation effect of the guidelines will be better, and vice versa.

## Conclusion

Improving methodology quality and strengthening clinical evidence is essential in the future guideline development in a range of disciplines for improving the treatment effectiveness of people with peri-implantitis. And there is a lack of integrated guidelines in the case of the COVID-19 pandemic.

## Electronic supplementary material

Below is the link to the electronic supplementary material.

**Supplementary material part 1**. Literature Search Strategies. **Supplementary material part 2**. A composite grading system for ranking recommendations in guidelines of COVID-19. **Supplementary material part 3**. PRISMA 2009 Checklist. **Supplementary table 1**. General characteristics of eligible guidelines. **Supplementary table 2**. AGREE II domain scores of included guidelines and overall assessment.

## Data Availability

The datasets used and/or analysed during the current study available from the corresponding author on reasonable request.

## Abbreviations and acronyms

CPGs: Clinical practice guidelines
AGREE: Appraisal of Guidelines for Research and Evaluation
PRISMA: Preferred Reporting Items for Systematic Reviews and Meta-Analyses
SPT: Support periodontal treatment
CBL: Crestal bone loss
