# Correction to: Critical review on quality of methodology and recommendations of clinical practice guidelines for peri-implantitis

**DOI:** 10.1186/s12903-023-02968-2

**Published:** 2023-05-03

**Authors:** Qiaofeng Yin, Jingyi Liang, Yuhang Zhang, Canxiong Chen, Weiming Yu, Xiaoyi Wang, Jianxin Ji

**Affiliations:** 1 Department of Stomatology, The Seventh Affiliated Hospital, Southern Medical University, 28 Desheng Liguan Road, Foshan, 528244 Guangdong P. R. China; 2 Department of Stomatology, The First Affiliated Hospital of Guangzhou Medical University, 151 Yanjiang Road, Guangzhou, 510120 Guangdong P. R. China; 3 National Center for respiratory medicine, state Key Laboratory of Respiratory Disease & National Clinical Research Center for Respiratory Disease, Guangzhou Institute of Respiratory Health, The First Affiliated Hospital of Guangzhou Medical University, Guangzhou, 510120 Guangdong P. R. China

**Correction: BMC Oral Health (2023) 23:189**

10.1186/s12903-023-02904-4

In this article, the author unit was marked incorrectly (affiliations 1 and 2 should be flipped). This has been corrected with this correction.

The original article has been corrected.

