# Supplementary material for: Critical review on quality of methodology and recommendations of clinical practice guidelines for peri-implantitis
Source: BMC Oral Health. 2023 Mar 31;23:189. doi: 10.1186/s12903-023-02904-4 (PMC10064959; doi:10.1186/s12903-023-02904-4)
Supplement: Supplementary file 1 — Supplementary material part 1. Literature Search Strategies. Supplementary material part 2. A composite grading system for ranking recommendations in guidelines of COVID-19. Supplementary material part 3. PRISMA 2009 Checklist. Supplementary table 1. General characteristics of eligible guidelines. Supplementary table 2. AGREE II domain scores of included guidelines and overall assessment. [file 12903_2023_2904_MOESM1_ESM.docx]

**Supplementary material**

**Supplementary material part 1. Literature Search Strategies**

**(1) MEDLINE (PubMed) Search Strategy**

#1 “Peri-implantitis” [Mesh Terms] (1,587)

#2 “Peri-implantitis” [Title/Abstract] (2,769)

#3 “Periimplantitis” [Title/Abstract] (2,683)

#4 “peri-implant disease*” [Title/Abstract] (658)

#5 Guideline [Publication Type] (36,232)

#6 guid* [Title/Abstract] (894,685)

#7 consensus* [Title/Abstract] (182,058)

#8 recommendat* [Title/Abstract] (293,506)

#9 best practice* [Title/Abstract] (30,673)

#9 #1 OR #2 OR #3 OR #4 (7,697)

#10 #5 OR #6 OR #7 OR #8 OR #9 (1,271,632)

#11 #9 AND # 10 (351)

**(2) Cochrane Library Search Strategy**

#1 MeSH descriptor: [Peri-implantitis] explode all trees (195)

#2 (Peri-implantitis OR Periimplantitis):ti,ab,kw (399)

#3 (Peri-implant disease*):ti,ab,kw (31)

#4 ("guid*" OR "consensus*" OR "recommendat*" OR "best practice*" OR "Clinical practice guideline*"):ti,ab,kw (118414)

#5 {OR #2-#4} AND #1 (46)

**(3) Web of science**

(TS=("Peri-implantitis" OR "Periimplantitis" OR "Peri-implant disease*")) AND TS=("guid*" OR "consensus*" OR "recommendat*" OR "best practice*" OR "CPG" OR "CPGs") (11)

**(4) Gray Literature Search**

1. Search Terms: (guideline) AND ("Peri-implantitis" OR "Periimplantitis" OR "Peri-implant disease*")
2. Sources:
3. Webpages of relevant organizations and journal
4. American Dental Association（0）
5. International Team for Implantology（0）
6. FDI World Dental Federation(0)
7. International College of Dentists(0)
8. America International Dental College(9)
9. Clinical Oral Implants Research (COIR)（0）
10. Clinical Implant Dentistry and Related Research (CIDRR)（0）
11. European Journal of Oral Implants (EJOI)（0）
12. The International Journal of Oral and Maxillofacial Implants (JOMI)（0）
13. Journal of Oral Implantology（0）
14. Implant Dentistry(0)
15. Grey database
16. [World Health Organization (WHO) Publications-](https://www.who.int/emergencies/diseases/novel-coronavirus-2019/technical-guidance) WHO guideline(0)
17. [National Institute for Health and Clinical Excellence (NICE)](http://www.nice.org.uk/) (0)
18. [Scottish Intercollegiate Guidelines Network (SIGN)](http://www.sign.ac.uk/index.html) (0)
19. [Guidelines International Network (G-I-N)](http://www.g-i-n.net/library/international-guidelines-library) (2)
20. Turn Research into Practice（TRP）（25）
21. BMJ best practice（0）
22. National Guidelines Clearinghouse(0)
23. Scottish Dental Clinical Effectiveness Programme(SDCEP)(0)
24. Citation index or other web sites (5)

**Supplementary material part 2. A** **composite grading system for ranking recommendations in guidelines of COVID-19**

| **Category** | **Grade** | **Definition** |
| --- | --- | --- |
| **Quality of evidence** | **High** | Randomized controlled trials without important limitations, or meta-analysis |
|  | **Moderate** | Randomized controlled trials with important limitations, or upgraded observational studies |
|  | **Low** | Non-randomized studies, cohort or case-control studies, case series |
|  | **Very low** | Expert opinion |
|  |  |  |
| **Strength of recommendation** | **Strong** | Recommendation can apply to most patients in many circumstances |
|  | **Weak** | The best action may differ depending on circumstances or patients’ or societal values |
|  | **Ungraded** | Insufficient evidence on which to formulate a recommendation. The advantages and disadvantages of the procedure/treatment are equivalent. |

| **Supplementary material part 3. PRISMA 2009 Checklist**   \| **Section/topic** \| **#** \| **Checklist item** \| **Reported on page #** \| \| --- \| --- \| --- \| --- \| \| **TITLE** \| \| \|  \| \| Title \| 1 \| Identify the report as a systematic review, meta-analysis, or both. \| Page 1 \| \| **ABSTRACT** \| \| \|  \| \| Structured summary \| 2 \| Provide a structured summary including, as applicable: background; objectives; data sources; study eligibility criteria, participants, and interventions; study appraisal and synthesis methods; results; limitations; conclusions and implications of key findings; systematic review registration number. \| Page 3 \| \| **INTRODUCTION** \| \| \|  \| \| Rationale \| 3 \| Describe the rationale for the review in the context of what is already known. \| Page 5 \| \| Objectives \| 4 \| Provide an explicit statement of questions being addressed with reference to participants, interventions, comparisons, outcomes, and study design (PICOS). \| Page 5 \| \| **METHODS** \| \| \|  \| \| Protocol and registration \| 5 \| Indicate if a review protocol exists, if and where it can be accessed (e.g., Web address), and, if available, provide registration information including registration number. \| Page 5 \| \| Eligibility criteria \| 6 \| Specify study characteristics (e.g., PICOS, length of follow-up) and report characteristics (e.g., years considered, language, publication status) used as criteria for eligibility, giving rationale. \| Page 5 \| \| Information sources \| 7 \| Describe all information sources (e.g., databases with dates of coverage, contact with study authors to identify additional studies) in the search and date last searched. \| Page 5 \| \| Search \| 8 \| Present full electronic search strategy for at least one database, including any limits used, such that it could be repeated. \| Supplementary material part 1 \| \| Study selection \| 9 \| State the process for selecting studies (i.e., screening, eligibility, included in systematic review, and, if applicable, included in the meta-analysis). \| Page 5 \| \| Data collection process \| 10 \| Describe method of data extraction from reports (e.g., piloted forms, independently, in duplicate) and any processes for obtaining and confirming data from investigators. \| Page 5 \| \| Data items \| 11 \| List and define all variables for which data were sought (e.g., PICOS, funding sources) and any assumptions and simplifications made. \| Page 5 \| \| Risk of bias in individual studies \| 12 \| Describe methods used for assessing risk of bias of individual studies (including specification of whether this was done at the study or outcome level), and how this information is to be used in any data synthesis. \| Page 5 \| \| Summary measures \| 13 \| State the principal summary measures (e.g., risk ratio, difference in means). \| Page 6 \| \| Synthesis of results \| 14 \| Describe the methods of handling data and combining results of studies, if done, including measures of consistency (e.g., I^2^) for each meta-analysis. \| NA \|  \| **Section/topic** \| **#** \| **Checklist item** \| **Reported on page #** \| \| --- \| --- \| --- \| --- \| \| Risk of bias across studies \| 15 \| Specify any assessment of risk of bias that may affect the cumulative evidence (e.g., publication bias, selective reporting within studies). \| Page 8 \| \| Additional analyses \| 16 \| Describe methods of additional analyses (e.g., sensitivity or subgroup analyses, meta-regression), if done, indicating which were pre-specified. \| NA \| \| **RESULTS** \| \| \|  \| \| Study selection \| 17 \| Give numbers of studies screened, assessed for eligibility, and included in the review, with reasons for exclusions at each stage, ideally with a flow diagram. \| Page 8 \| \| Study characteristics \| 18 \| For each study, present characteristics for which data were extracted (e.g., study size, PICOS, follow-up period) and provide the citations. \| Page 8  Supplementary table 1. \| \| Risk of bias within studies \| 19 \| Present data on risk of bias of each study and, if available, any outcome level assessment (see item 12). \| Page 8 \| \| Results of individual studies \| 20 \| For all outcomes considered (benefits or harms), present, for each study: (a) simple summary data for each intervention group (b) effect estimates and confidence intervals, ideally with a forest plot. \| Supplementary table 1 \| \| Synthesis of results \| 21 \| Present results of each meta-analysis done, including confidence intervals and measures of consistency. \| NA \| \| Risk of bias across studies \| 22 \| Present results of any assessment of risk of bias across studies (see Item 15). \| NA \| \| Additional analysis \| 23 \| Give results of additional analyses, if done (e.g., sensitivity or subgroup analyses, meta-regression [see Item 16]). \| NA \| \| **DISCUSSION** \| \| \|  \| \| Summary of evidence \| 24 \| Summarize the main findings including the strength of evidence for each main outcome; consider their relevance to key groups (e.g., healthcare providers, users, and policy makers). \| Page 9-11 \| \| Limitations \| 25 \| Discuss limitations at study and outcome level (e.g., risk of bias), and at review-level (e.g., incomplete retrieval of identified research, reporting bias). \| Page 11 \| \| Conclusions \| 26 \| Provide a general interpretation of the results in the context of other evidence, and implications for future research. \| Page 12 \| \| **FUNDING** \| \| \|  \| \| Funding \| 27 \| Describe sources of funding for the systematic review and other support (e.g., supply of data); role of funders for the systematic review. \| Page 19 \|   **Supplementary table 1. General characteristics of eligible guidelines** | | | | | | | | | | | | | |
| --- | --- | --- | --- | --- | --- | --- | --- | --- | --- | --- | --- | --- | --- | --- | --- | --- | --- | --- | --- | --- | --- | --- | --- | --- | --- | --- | --- | --- | --- | --- | --- | --- | --- | --- | --- | --- | --- | --- | --- | --- | --- | --- | --- | --- | --- | --- | --- | --- | --- | --- | --- | --- | --- | --- | --- | --- | --- | --- | --- | --- | --- | --- | --- | --- | --- | --- | --- | --- | --- | --- | --- | --- | --- | --- | --- | --- | --- | --- | --- | --- | --- | --- | --- | --- | --- | --- | --- | --- | --- | --- | --- | --- | --- | --- | --- | --- | --- | --- | --- | --- | --- | --- | --- | --- | --- | --- | --- | --- | --- | --- | --- | --- | --- | --- | --- | --- | --- | --- | --- | --- | --- | --- | --- | --- | --- | --- | --- | --- | --- | --- | --- | --- | --- | --- | --- | --- | --- | --- | --- | --- | --- | --- | --- | --- | --- | --- | --- | --- | --- | --- | --- | --- | --- | --- | --- | --- | --- |
| Title | Issuing society Full name | Acronym of the guideline | Date of publication | Region | Type of guidelines | Type of publication | Development method | Strength of Recommendation | Quality of Evidence | Grading system | Version | Developers | Number of_developed_organization |
| Peri-implantdiseases:Consensus Report of the Sixth European Workshop on Periodontology | the European Workshop on Periodontology | EWP(1) | 2008 | International | Not only Management | others | EB | No | No | - | First | Medical society | 1 |
| Statements from the Estepona Consensus Meeting on Peri-implantitis, February 2–4, 2012 | Tomas Albrektsson et al. | Albrektsson et al. | 2012 | International | Not only Management | Others | Non-EB | No | No | - | First | Medical society | 1 |
| Peri-implant tissue destruction. The Third EAO Consensus Conference 2012 | European Association for Osseointegration | EAO(1) | 2012 | International | Not only Management | others | EB | No | No | - | First | Medical society | 1 |
| Consensus statements and clinical recommendations for prevention and management of biologic and technical implant complications | International Team for Implantology | ITI | 2014 | International | Not only Management | Guideline | EB | No | No | - | First | Medical society | 1 |
| Primary and Secondary Prevention of Periodontal and Peri-Implant Diseases | European Workshop on  Periodontology | EWP(2) | 2015 | International | Prevention | others | EB | No | No | - | First | Medical society | 1 |
| Recommendations on the clinical application of air polishing for the management of peri-implant mucositis and peri-implantitis | A group of international experts in dental implant | Schwarz et al. | 2016 | International | Management | others | EB | No | No | - | First | Medical society | >1 |
| The 1st Baltic Osseointegration Academy and Lithuanian University of Health Sciences  Consensus Conference 2016. Summary and Consensus Statements: Group I - Peri-Implantitis Aetiology, Risk Factors and Pathogenesis | Baltic Osseointegration Academy and Lithuanian University of  Health Sciences | BOA&LUHS(1) | 2016 | International | Prevention | others | EB | No | No | - | First | Medical society | >1 |
| The 1st Baltic Osseointegration Academy and Lithuanian University of  Health Sciences Consensus Conference 2016. Summary and Consensus  Statements: Group III - Peri-Implantitis Treatment | Baltic Osseointegration Academy and Lithuanian University of  Health Sciences | BOA&LUHS(2) | 2016 | International | Management | others | EB | No | No | - | First | Medical society | >1 |
| The 1st Baltic Osseointegration Academy and Lithuanian University of  Health Sciences Consensus Conference 2016. Summary and Consensus  Statements: Group II - Peri-Implantitis Diagnostics and Decision Tree | Baltic Osseointegration Academy and Lithuanian University of  Health Sciences | BOA&LUHS(3) | 2016 | International | Not only Management | others | EB | No | No | - | First | Medical society | >1 |
| Consensus Report: Peri-implant Diseases and Conditions | e American Academy of Periodontology and the European Federation of Periodon tology | AAP&EFP | 2018 | International | Diagnosis | others | Non-EB | Yes | Yes | - | First | Medical society | >1 |
| Antimicrobial Photodynamic Therapy for the Treatment of  Periodontitis and Peri-Implantitis: An American Academy of  Periodontology Best Evidence Review | American Academy of Periodontology | AAP(1) | 2018 | International | Management | others | EB | No | No | - | First | Medical society | >1 |
| American Academy of Periodontology Best Evidence Consensus Statement on  the Efficacy of Laser Therapy Used Alone or as an Adjunct to Non-Surgical and  Surgical Treatment of Periodontitis and Peri-Implant Diseases | American Academy of Periodontology | AAP(2) | 2018 | International | Management | others | EB | No | No | - | First | Medical society | 1 |
| Drugs and diseases: Summary and consensus statements of  group 1. The 5th EAO Consensus Conference 2018 | European Association for Osseointegration | EAO(2) | 2018 | International | Not only Management | others | EB | No | No | - | First | Medical society | 1 |
| Surgical treatment of peri-implantitis – Consensus report of working group 4 | FDI WORLD DENTAL FEDERATION | FDI(1) | 2019 | International | Management | Others | EB | No | No | - | First | Medical society | 1 |
| Diagnosis and non-surgical treatment of peri-implant diseases and maintenance care of patients with dental implants— Consensus report of working group 3 | FDI World Dental Federation | FDI(2) | 2019 | International | Not only Management | others | EB | No | No | - | First | Medical society | 1 |

**Supplementary table 2. AGREE II domain scores of included guidelines and overall assessment**

| **Guidelines** | ***Scope and Purpose*** | ***Stakeholder Involvement*** | ***Rigor of Development*** | ***Clarity and Presentation*** | ***Applicability*** | ***Editorial Independence*** | ***Overall Assessment*** |
| --- | --- | --- | --- | --- | --- | --- | --- |
| EWP(1) | 39 | 24 | 13 | 56 | 29 | 75 | Recommended with modification |
| Albrektsson et al. | 18 | 13 | 6 | 43 | 4 | 4 | Not recommended |
| EAO(1) | 67 | 44 | 16 | 69 | 21 | 58 | Recommended with modification |
| ITI | 56 | 51 | 7 | 56 | 25 | 58 | Recommended with modification |
| EWP(2) | 33 | 7 | 9 | 33 | 13 | 60 | Not recommended |
| Schwarz et al. | 50 | 39 | 10 | 50 | 17 | 33 | Recommended with modification |
| BOA&LUHS(1) | 49 | 25 | 38 | 44 | 5 | 48 | Recommended with modification |
| BOA&LUHS(2) | 54 | 25 | 38 | 44 | 5 | 48 | Recommended with modification |
| BOA&LUHS(3) | 50 | 22 | 39 | 44 | 5 | 46 | Recommended with modification |
| AAP&EFP | 33 | 25 | 9 | 39 | 17 | 54 | Not recommended |
| AAP(1) | 61 | 22 | 36 | 50 | 20 | 63 | Recommended with modification |
| AAP(2) | 50 | 21 | 27 | 33 | 21 | 33 | Recommended with modification |
| EAO(2) | 32 | 22 | 19 | 50 | 13 | 17 | Not recommended |
| FDI(1) | 67 | 44 | 15 | 61 | 21 | 67 | Recommended with modification |
| FDI(2) | 61 | 33 | 15 | 39 | 10 | 65 | Recommended with modification |
| Total # | 49 (18,67) | 28 (7,51) | 20 (6,39) | 47(33,69) | 14 (4,25) | 47 (4,67) |  |

*: The full name of the abbreviation is as same as those in Table S1.

#: Data are presented as mean and range.
